# Modeling Temporal Interaction Dynamics in Organizational Settings

**DOI:** 10.1007/s10869-017-9506-9

**Published:** 2017-08-26

**Authors:** Nale Lehmann-Willenbrock, Joseph A. Allen

**Affiliations:** 1 University of Amsterdam, Work and Organizational Psychology, P.O. Box 15919, 1001 NK Amsterdam, The Netherlands; 2 University of Nebraska at Omaha, Omaha, USA

**Keywords:** Behavioral observations, Interaction analysis, Temporal patterns, Pattern analysis, Software options

## Abstract

Most workplace phenomena take place in dynamic social settings and emerge over time, and scholars have repeatedly called for more research into the temporal dynamics of organizational behavior. One reason for this persistent research gap could be that organizational scholars are not aware of the methodological advances that are available today for modeling temporal interactions and detecting behavioral patterns that emerge over time. To facilitate such awareness, this *Methods Corner* contribution provides a hands-on tutorial for capturing and quantifying temporal behavioral patterns and for leveraging rich interaction data in organizational settings. We provide an overview of different approaches and methodologies for examining temporal interaction patterns, along with detailed information about the type of data that needs to be gathered in order to apply each method as well as the analytical steps (and available software options) involved in each method. Specifically, we discuss and illustrate lag sequential analysis, pattern analysis, statistical discourse analysis, and visualization methods for identifying temporal patterns in interaction data. We also provide key takeaways for integrating these methods more firmly in the field of organizational research and for moving interaction analytical research forward.

This is the tenth paper in the *Methods Corner* series of the *Journal of Business and Psychology*. Previous works in this series have focused on a range of methodological issues and applications in business and managerial psychology, including tests of mediation (MacKinnon, Coxe, & Baraldi, 2012), the implementation of structural equation modeling for meta-analysis (Landis, 2013), the use of moderator models and analysis (Dawson, 2014), the analysis of historical data in organizational research (Zickar, 2015), the use of latent variable confirmatory factor analysis for addressing common method variance (Williams & McGonagle, 2016), and most recently, the precision and utility of mixed-effects models (Bliese, Maltarich, & Hendricks, 2017). The current paper adds a new perspective to the series by highlighting different ways to study and model temporal interaction dynamics in organizational settings.

Organizational scholars increasingly appreciate the value of focusing on behavior and modeling temporal behavioral contingencies, as indicated by several conceptual and theoretical works in the team process as well as the leadership literature (e.g., Cronin, Weingart, & Todorova, 2011; DeRue, 2011; Dinh, Lord, Gardner, Meuser, Liden, & Hu, 2014; Herndon & Lewis, 2015; Leenders, Contractor, & DeChurch, 2016; Waller, Okhuysen, & Saghafian, 2016). Yet, empirical efforts at addressing the calls that are put forth in these conceptual papers remain sparse. One reason could be that organizational scholars are not aware of the methodological advances that are available today for modeling social dynamics in behavioral interactions. To address this gap, this *Methods Corner* paper highlights social interaction analysis as a rich temporal behavioral approach for studying workplace phenomena such as team process dynamics and leader-follower interactions.

Rather than studying (single or multiple) snapshots of behavior, research on actual interactions as they unfold over time can generate insights into the complex social dynamics at the core of many organizational phenomena, as most employee behaviors are embedded in dynamic social contexts (e.g., Johns, 2006; Mehra, Kilduff, & Brass, 2001; Porath, Spreitzer, Gibson, & Garnett, 2012; Spreitzer, Sutcliffe, Dutton, Sonenshein, & Grant, 2005). The goal is to get closer to the phenomena of interest, to investigate the actual behaviors that we are trying to explain, and to understand the temporal dynamics that surround them. For example, instead of static descriptions of a leader’s overall style, behavioral interaction research can yield much more specific answers about what, when, and how a leader needs to communicate in order to motivate their team toward a particular goal.

Providing some answers to the repeated calls for more dynamic research on team processes (e.g., Kozlowski, 2015) and on leader-follower interactions (e.g., Dinh et al., 2014), a number of recent studies have adopted a temporal approach and begun to study actual behavioral interactions in the workplace (e.g., Meinecke, Lehmann-Willenbrock, & Kauffeld, 2017; Paletz, Chan, & Schunn, 2016; Zijlstra, Waller, & Phillips, 2012). In particular, a small but growing research base investigates actual behaviors and behavioral patterns embedded in workplace interactions. We highlight a number of these advances in the literature as exemplary applications of pattern analytical methods. In keeping with the emphasis of our paper, focus on those studies that have provided insights into socially embedded behaviors or interaction patterns, rather than Likert-type frequency scales or static counts of behavior (for examples of the latter, see Detert & Burris, 2007; Hirst, van Knippenberg, & Zhou, 2009; Madrid, Totterdell, & Niven, 2016; Totterdell, 2000; among many others, including some of our own work). Moreover, we highlight only those studies that have offered insights based on actual workplace populations, rather than undergraduate or MBA students and their ad hoc interactions in the laboratory (among a multitude of examples, see Aggarwal & Woolley, 2013; Hambley, O’Neill, & Kline, 2007; Nahrgang, DeRue, Hollenbeck, Spitzmuller, Jundt, & Ilgen, 2013).

In the form of a detailed methodological tutorial, we briefly review the basic approach to observing and analyzing social interactions in the workplace and then point out different analytical strategies for exploring temporal dynamics in detail. We provide an overview of available methodologies for quantifying temporal interaction patterns and discuss the type of research questions that can be addressed by each method as well as available software options. Specifically, we discuss lag sequential analysis, pattern analysis, and statistical discourse analysis for quantifying emergent behavioral patterns and testing hypotheses at the behavioral event level. Moreover, we also highlight a number of more exploratory visualization methods for investigating temporal interaction patterns. We hope that this paper will inspire future research to take a more dynamic stance when studying interactions at work. Our aim here is to lay the groundwork for much needed empirical advances in terms of understanding social dynamics in in the workplace.

## Conceptualizing Behavior as Actual Behavior

In 2011, Cronin et al. stated, “We hope that a review of the group dynamics literature in 2021 will celebrate our coming empirical accomplishments rather than lament a lack of them” (p. 571). Their largest concern was the lack of progress in analyzing social dynamics in teams as behavior unfolds over time. The hope was that increased research effort, technological advances, statistical methodological discoveries, and the incorporation of time in research questions and design would help bring the modeling of temporal interaction dynamics forward. A key consideration in modeling behavior through time is conceptualizing and capturing behavioral phenomena as actual behavior, rather than relying on post hoc perceptions of behavior (e.g., surveys) or the filtered interpretations of such behavior (e.g., anecdotal observations by researchers; Baumeister, Vohs, & Funder, 2007).

Actual behavior concerns the observable movements, interactions, communications, and so forth that individuals and groups actually engage in (e.g., Baumeister et al., 2007). For example, when a leader seeks to motivate their team, knowing the actual communicative behaviors they should engage in within the interaction stream (i.e., which actual behavior should be executed at which particular point in time) seems essential and more practical than just knowing the post hoc perceptions of a leader’s overall behavior. The benefit of studying actual behavioral markers of phenomena such as team coordination and problem solving or social influence between leaders and followers is that the obtained behavioral data are closer to the phenomena of interest, both conceptually and methodologically (e.g., Baumeister et al., 2007). For example, when studying humor in group or team interactions, humor and laughter occurrences are key behavioral markers (Lehmann-Willenbrock & Allen, 2014). Thus, a statement intended to be humorous (and often followed by laughter) is a behavioral marker that is conceptually and temporally closer to the phenomenon of humor than the post-interaction survey response that the interaction was humorous.

More specifically, Baumeister et al., (2007) articulate and lament the fact that much of psychological science focuses on “self-reports and finger movements” rather than actual behavior. With industrial/organizational psychology and other organizational sciences interested in the behavior of people, it is remarkable that so much of the recent work in this area continues to rely on surveys where researchers get the filtered, interpreted reflection of previous behavior rather than actually observing behavior as it happens in real time (Agnew, Carlston, Graziano, & Kelly, 2009). According to Agnew et al., psychological science investigates inter-individual (e.g., social cognition and individual differences) and intra-individual (e.g., social relationships and group dynamics) processes, that should not be divorced from or devoid of behavior and observation. Yet, so much of modern psychological science continues to depend upon interpretation, absent of behavioral referent.

Frankly, this returns, to some extent, to the classic debate between behaviorism (e.g., Skinner, 1974) and cognitivism (Gardner, 2008). In an oversimplified statement, behaviorism asserts that researchers should focus only on the external behaviors of individuals while cognitivism asserts that they should focus only on individuals’ internal processing and thought. Both are essential and both have a long history of research, investigation, and monumental discovery. For example, the discovery of operant conditioning (Skinner, Skinner, Psychologue, Skinner, & Skinner, 1972) is a result of a behavioral approach, whereas Premack’s Theory of Mind (Premack & Woodruff, 1978) is directly a function of a transition to the cognitive approach.

Given what we have stated, one might start to believe that we are advocating for a closer look at behavior at the expense of cognition or interpretation and so forth. To the contrary, we advocate investigating temporal interaction from an inclusive perspective that includes both behavior and cognition. For example, Lehmann-Willenbrock, Meyers, Kauffeld, Neininger, and Henschel (2011) investigated verbal interaction sequences in relation to group mood. Group mood is inherently an internal affective process that has behavioral manifestations. Thus, the investigation of temporal group dynamics may and often does include both behavior and internal cognitive processes. In many cases, however, when we want to understand interactions between individuals or within groups, behavioral data is often better and more appropriate than the post-interaction interpretations reported on a survey. Thus, without taking away the need or appropriateness of surveys or other methods, we focus on the need and the call by others (e.g., Baumeister et al., 2007) to study actual behavior in groups and among individuals in dynamic social interaction.

## Tutorial: How to Investigate Dynamic Temporal Interactions

When considering the use of methods for observing and analyzing temporal interaction patterns, scholars need to make several key decisions. We summarize these steps in Table 1, and the remainder of this paper will follow this structure accordingly. Specifically, the table summarizes the five major steps to doing dynamic temporal interaction research. First, specifying the interaction context and overarching research question. Second, specifying the procedure for data gathering, unitizing, and coding. Third, selecting software to support the coding and subsequent analyses. Fourth, selecting a pattern analytical method for understanding the interaction observed. Fifth, running analyses appropriate for the research question and interpreting the results. With each of these steps, we provide key action items/questions in the table and provide interpretation as well as additional details in this text. We also recommend additional resources, including possible software applications, for interested researchers.

**Table 1:** Key decision points and considerations for setting up interaction analytical research

Key decision	Action items and questions to address	Further details
Specifying the interaction context and overarching research question	• Which behaviors suggest the interaction of interest is occurring? • Where are these interactions most likely to occur? (i.e., meetings, interviews) • How will the interactions impact the individuals/teams in terms of (a) within context processes and (b) outcomes?	See Table 2 for examples
Specifying the procedure for data gathering, unitizing, and coding	• How should the data be recorded? Audio? Video? Both? Other? • What are the available coding schemes? Do they fit the question/interaction of interest? If not, how to create a coding scheme? • What is the unit of interest within the interaction? Utterance versus pattern versus other	E.g., Meinecke and Lehmann-Willenbrock (2015)
Selecting software to support the coding nd subsequent analyses	• Which functions should be included? • Consider both quantitative analytical functions and visualization options • How many licenses are needed? E.g., at least two licenses to equip two coders who can work simultaneously	See Table 3
Selecting a pattern analytical method	• Which type of research question needs to be addressed? E.g., “Do problem analysis statements trigger solutions?” • Which analytical approach is needed for addressing this question? E.g., How often do solutions follow problem analysis statements in the data? Is this behavioral sequence statistically meaningful? ➔ Select lag sequential analysis	See Table 4
Running analyses and interpreting the results	• What do significance tests tell us about the interaction pattern? • How to move from counting patterns to predicting patterns and outcomes of interactions?	E.g., Bakeman and Quera (2011), for lag sequential analysis; Magnusson (2000), for pattern analysis; Chiu and Lehmann-Willenbrock (2016), for Statistical Discourse Analysis

For the first step, previous research, particularly in the groups literature, emphasizes the importance of studying communicative behaviors in order to understand what actually happens in groups (e.g., Bonito & Sanders, 2011; Gouran, 1999; Gouran & Hirokawa, 1996; Jarboe, 1999; Meyers & Brashers, 1999; Pavitt, 1993, 1999; Poole, 1999). In this tutorial, we focus on one example, team problem solving, and walk through the steps in the process to analyze dynamic temporal interactions. There are, however, many research questions both related to groups and teams as well as leadership, among other contexts, for which this type of analysis can be used. A few examples are provided in Table 2. Specifically, Table 2 provides an overview of specific research topics or phenomena, potential verbal/nonverbal behaviors of interest in the context of each phenomenon, the respective unitizing decision, and a suggested method for data gathering.

**Table 2:** Example research topics for modeling dynamic temporal interactions

Phenomenon of interest	Behavioral indicators	Unitizing decision	Data gathering
Team problem solving	Specific verbal behaviors: Stating a problem; stating an idea; asking a question (e.g., Lehmann-Willenbrock, Allen, & Meinecke, 2014)	Sense units (Bales, 1950)	Video recorded team interactions
Leader-follower relationships	Specific verbal and nonverbal behaviors: Supportive statements; sharing ideas; encouragement; expressing concern	Sense units and nonverbal cues (e.g., Nowicki & Duke, 1994)	Video and/or audio recorded dyadic interactions
Group mood	Nonverbal behaviors: Frowning; smiling; other facial expressions; hand gestures posture (e.g., Krauss, Chen, & Chawla, 1996; Lehmann-Willenbrock et al., 2011)	e.g., 2-min segments (Barsade, 2002)	Video recorded group interactions
Inspirational leadership in groups	Specific verbal behaviors: Identified positive statements based upon theory; encouragement/supportive socioemotional statements; solution-oriented statements (e.g., Lehmann-Willenbrock et al., 2015)	Sense units (Bales, 1950)	Video recorded group (i.e. leader-follower) interactions
Group consensus	Specific verbal and nonverbal behaviors: Supportive statements; agreement statements; procedural statements; nods; smiling	Sense units and focused segments (e.g., final decision moments of a group meeting)	Video recorded group interactions

For example, a researcher who studies group mood might be interested in understanding the nonverbal behaviors that indicate changes in group mood. From a theoretical stance, group mood develops because team members respond to one another’s affective expressions (for an overview, see Barsade & Knight, 2015). Hence, to pinpoint the phenomenon of group mood, we need to study group members’ visible behavioral expressions of affect. These would be indexed by observing and coding group members’ instances of smiling, frowning, other facial expressions, hand gestures, posture, and so forth. However, because group mood is a dynamic process that may change from moment to moment within the group interaction, the stream of interaction may be segmented into temporal units (e.g., 2-min segments; Barsade, 2002) and comparisons made across segments. In order to do this, video-recorded group interaction would likely be essential. The table provides several additional examples similar to group mood just described here. For additional detail on unitizing decisions and the coding process, as well as helpful additional examples, see Meinecke & Lehmann-Willenbrock, 2015; Chiu & Lehmann-Willenbrock, 2016.

### Identifying Relevant Behaviors

As mentioned, we will use the example of team problem solving. Our research question is “how does team problem solving unfold within team interactions?”. With this research question defined, we must decide on the variable or set of variables that can suitably operationalize this phenomenon at the behavioral level. That is, unlike other methods (e.g., longitudinal survey designs) where we seek participant observations or interpretations of behavior (e.g., how many problem statements did you make?), we want to identify the actual behaviors that the researcher or independent coders can observe, count, quantify, and evaluate that are related to the phenomenon of interest. For example, in the case of team problem solving, we might be interested in the specific, observable verbal behaviors that are indicative of team problem solving, such as idea generation, problem statements, and solution statements (e.g., Kauffeld & Lehmann-Willenbrock, 2012). Further, the behaviors themselves are situated within the dialog and within the group dynamics such that some statements will be easier or harder to code for the specific behavior investigated. For example, perhaps the problem being solved is a parking issue at the corporate building. As the dialog unfolds, a problem statement might be very overt such as, “I think the real problem is parking administrators sold too many passes to our parking lot”. In other cases, the statement might be more situated within dialog such as, “I was driving around for an hour the other day looking for parking, and I think there are too few spaces and some slots are too large”. In this case, they never overtly state that “the problem is,” but they more subtly suggest that a problem is in the nature of the parking stalls.

### Defining Behavioral Units

The second step in interaction analytical research concerns the issues of data gathering, unitizing, and coding. We begin with unitizing as that impacts both how the data should be gathered and the appropriate coding scheme to use. When deciding on a unitizing rule for the research, the key question is whether the behavioral codes are assigned to either a behavioral event or a specific time interval (Bakeman & Quera, 2011). This so-called unitizing decision can be either deductive, or it may be inductive. A deductive approach to the unitizing decision typically requires an established coding scheme, where unitizing rules are clear prior to data gathering (e.g., interaction process analysis, Bales, 1950; or the act4teams coding scheme, Kauffeld & Lehmann-Willenbrock, 2012). Instead, an inductive approach to the unitizing decision would mean that interaction data is gathered and inspected before this decision is made. This can be the case when a research question or interaction context is entirely novel, such that published coding schemes cannot be applied to the data at hand.

Social interactions in the workplace, such as those occurring during regular team meetings or in a conversation between leaders and followers, are typically characterized by topic changes, participation shifts, dynamic speaker switches, or conversational turn-taking (e.g., Gibson, 2003, 2005; Lehmann-Willenbrock, Chiu, Lei, & Kauffeld, 2016; van Oortmerssen, van Woerkum, & Aarts, 2015). These changes occur at the level of minutes, seconds, and milliseconds rather than days or weeks. The decision how much a researcher needs to “zoom in” in order to establish the adequate timeframe will be driven by the research question. In our example, team problem solving, we are interested in behavioral events such as a problem statements rather than a segment of time. These statements might most easily be observed as turns of talk or when speakers switch, though not exclusively since a monolog could include a problem statement and a solution statement (e.g., Chiu, 2000). Using the rule that we are interested in complete speaker turns, we would then separate or “cut” the interaction stream such that a new behavioral unit is assigned whenever the speaker changes. It should be noted that the unitizing decision would differ across research topics as illustrated in Table 2. Unitizing according to turns of talk can be the method of choice for many research questions. For example, researchers have studied behavioral turns of talk when examining the way in which meeting attendees react to one another and shape the social network (Laapotti & Mikkola, 2016; Sauer & Kauffeld, 2013).

However, for many other research questions, speaker turns may not be fine-grained enough in terms of the behavioral units obtained. The segmentation of the interaction stream into individual behaviors may then need to be more fine-grained than segmenting simple speaker turns. To return to our earlier example, the researcher interested in problem solving in meetings may be well advised to separate smaller behavioral units in order to investigate the functionality of specific statements within the interaction. For example, within the same speaker turn, a meeting attendee may first suggest an idea and then offer a reason for proceeding with that idea immediately afterwards. Thus, instead of speaker turns, it is advisable to distinguish between “sense units” within a given turn (e.g., Bales, 1950) through the interaction flow.

In contrast to segmenting the interaction stream according to sense units or other behavioral events, a researcher may need to consider the duration of the behavioral unit of interest. Fixed time intervals instead of behavioral events may be required for some research questions in terms of coding. For example, as mentioned concerning Table 2, research on emotions and moods in groups and teams has investigated changes over the course of a meeting in group affect by coding a segment every 2 min (Barsade, 2002; Lei & Lehmann-Willenbrock, 2015; see also Waller, Zellmer-Bruhn, & Giambatista, 2002, for a similar unitizing approach). The decision how specific or broad such time intervals are should be driven by theory (e.g., existing assumptions about different team or leader behaviors in broader team phases; e.g., Morgeson, DeRue, & Karam, 2010) as well as empirical considerations. The latter may include initial observations from the recorded interaction data, such as how frequently teams typically change topic. For examples and additional discussion of unitizing decisions and time frames, see Bakeman & Quera, 2011.

### Coding Behavior

Once the unitizing rule is chosen, the researcher must decide how to code the behavioral units. “Coding” in this context means that every behavioral unit will be assigned to a behavioral category. Note that sometimes this is called “annotating” rather than “coding.” For our example, we have already identified three potential categories under which we would want to code the observed behaviors including idea statement (i.e., idea generation), solution statements, and problem statements. Due to these statements being embedded within a larger stream of interaction, it is advisable that the coding scheme is exhaustive thereby avoiding room for interpretation that will likely pose a threat to inter-rater reliability and to ease the coding procedure (Bakeman & Quera, 2011). In other words, a coder should be able to assign any unit that is selected or cut from the meeting interaction flow to a behavioral code within the coding scheme. For example, returning to our team problem solving concerning parking, in addition to making a problem statement (e.g., “I think the real problem is parking administrators sold too many passes to our parking lot”), a team member may say “yeah, I agree!”—an agreement statement which is not specific to our research question. Or someone on the team may say, “I agree, the process for purchasing permits does not consider the number of purchasers”, which both agrees with the problem statement and elaborates upon the problem. As such, even for our specific research question, it is appropriate and recommended to select or create a coding scheme that would code every behavioral unit, even if it does not necessarily pertain to the research question at hand. Leaving units uncoded can be problematic later in the research process, depending upon the analysis strategy chosen. Rather than leave them uncoded, behaviors that really do not fit any of the categories in a coding scheme might be coded as “no fit” or “other”.

### Gathering Behavioral Interaction Data

Once a decision is made about the phenomenon of interest, then determining the best method for capturing the interactions where the phenomenon occurs comes next. For our current research question and for those listed in Table 2, we decided to focus on video and audio recording for capturing the interactions. Of note, there are other modalities for behavioral observations, namely wearable sensors and other unobtrusive behavioral measures. Extracting and meaningfully interpreting such sensor data typically require the expertise of computer scientists. For an overview of possible approaches and interdisciplinary research opportunities, see Lehmann-Willenbrock, Hung, and Keyton (forthcoming). Yet, in terms of behavioral team interaction processes, much of the previous work relied on videotaped meeting interactions (e.g., Kauffeld & Lehmann-Willenbrock, 2012; Kauffeld & Meyers, 2009). Videos are particularly useful when analyzing group data because there are multiple people and tracking who is speaking when is essential for coding the interaction. As with our question, someone may make a problem statement, another person may elaborate on the problem, another may present a potential solution, followed by another proposing a new idea. In this case, video-recorded interactions is probably the easiest way to observe the movement from person to person, though some audio recording setups will allow for capturing this information (see also Dent, Brown, Dowsett, Tattersall, & Butow, 2005; Nicolai, Demmel, & Farsch, 2010). Note that one concern of research in this manner is how the video camera may change behavior, simply by being in the room. Previous research, however, shows that participants who are advised to ignore the camera fall into their regular routine, as indicated by behaviors such as telling jokes, or criticizing absent supervisors (e.g., Lehmann-Willenbrock & Kauffeld, 2010; see also Coleman, 2000; Herzmark, 1985; Penner, Orom, Albrecht, Franks, Foster, & Ruckdeschel, 2007). The positioning of the camera as well as the quality of the video/audio should also be considered when making decisions on how to best capture the interactions.

## Software Options

The third step in the process of doing temporal interaction research is selecting the appropriate software support. It should be noted that coding and analyzing interaction data does not necessarily have to be software-assisted. However, we highly recommend using professional software when working with video or audio data. This recommendation holds for our current example of studying team problem solving because of the complexity and volume of data to be coded. There are several software options available on the market, such as The Observer XT software (Noldus, Trienes, Hendriksen, Jansen, & Jansen, 2000) or INTERACT software (Mangold, 2010). Essentially, these software packages help segment a stream of behavior in a video or audio file into individual behavioral units, which can then be assigned a behavioral code. Hence, a major benefit of this software-assisted coding is the fact that it is no longer necessary to transcribe the verbal content of each person’s behavior on the video (or audio) file. Instead, researchers can directly assign a behavioral code such as “problem,” “solution,” or “question” to each behavior when it is cut out from the video stream. This facilitates the quantification of qualitative content data, while preserving the temporal embeddedness of each behavior within the interaction flow. Note that this unitizing functionality of software such as INTERACT or The Observer XT is not an automated process and a human coder is still required to hit the “start” and “stop” button and tell the software where to cut out each behavior from the video or audio file. In other words, the unitizing process is still largely dependent on human effort (a notable exception concerns cases where behavioral data can be automatically annotated; we will return to such cases in our outlook section).

One more point needs to be made regarding inter-rater reliability when using software to code live video. In this case, units are marked according to time rather than words. The smallest time units are usually frames per seconds; thus, it is nearly impossible for two coders to cut the video at the exact same time. A common procedure here is to construct clear unitizing rules and to employ just one trained unitizer to identify the units. Subsequently, other trained coders assign these identified units to a behavioral code from the coding scheme. Although this is an acceptable and useful procedure, there are times when unitizing and time segments are of interest to a research question. For example, one might be interested in the moment when a team’s atmosphere or general mood switched. In this case, the timing of the units could be of interest in determining at one point an independent rater indicates when the mood changed. Further, a flaw of this approach is the assumption that the trained unitizer is correct in the way that they unitize the data. At a minimum, a second trained unitizer should review the work of the other to ensure that the unitizing was done effectively. Though it is impossible to get agreement in the truest statistical sense, a second unitizer can verify that no behavioral units are inadvertently combined through sloppy unitizing or simply not hearing a statement by a quiet group member in the interaction stream.

Additionally, there are also newer and more advanced measures to calculate inter-rater reliability so that, for example, coders can simultaneously unitize (i.e., cut into segments) and code streams of behavior (see Bakeman, Quera, & Gnisci, 2009, for an example). Bakeman et al. (2009) developed the GSEQ software system (see Table 3) that allows the calculation of agreement between raters when they both unitize and code independently. Thus, it is possible to get both agreement in terms of unitizing as well as the coded behavior. However, it is possible in some cases to have agreement on coded behavior and not on the unitizing. In this case, one must investigate the source of the lack of agreement. Is it a function of different unitizing strategies or simply an accumulation of tiny frames-per-second differences? Further, if coded behavior agreement is not achieved, standard categorical analysis procedures for training and retraining coders should be followed (Krippendorff, 2004).

**Table 3:** Software for quantifying temporal interaction patterns

Software	Analysis and functionality	Cost
GSEQ (provider: Richard Bakeman and Vicenç Quera) ﻿Available at http://www2.gsu.edu/~psyrab/gseq/	Analysis of sequential observational data (no event logging; i.e., data should already be coded) Descriptive statistics: frequencies, joint frequencies, rates, durations, and proportions of observed behavior Adjusted residuals, chi-squares, Yule’s Q, and odds ratios Event- and time-based inter-rater agreement	Free
GridWare (Lamey, Hollenstein, Lewis, & Granic, 2004) Available at http://statespacegrids.org	State space grids	Free
INTERACT (provider: Mangold International)	Event logging and coding directly from video or audio files; extensive options for editing and refining codes Extensive descriptive statistics: e.g., frequencies, duration, percentages Inter-rater reliability: Cohen’s Kappa and ICC Lag sequential analysis Pattern analysis State space grids Plug-in options: Integrated programming language for creating import/ export and analysis routines (syntax files that can be shared among users)	Price quote for academic use: EUR 6200 (USD 6587) for a full license that includes lag sequential analysis and pattern analysis
The Observer XT(provider: Noldus Information Technology)	Event logging and coding directly from video or audio files Extensive descriptive statistics: e.g., frequencies, duration, percentages Inter-rater reliability: Cohen’s Kappa Lag sequential analysis Plug-in options: Software development kit for connecting custom software components and data interfaces	Price quote for academic use: EUR 4900 (USD 5186) for a license including the 2 Media Module and the Advanced Analysis Module for lag sequential analysis

Software options are listed in alphabetical order. Price quotes obtained in 2017 via personal inquiry at the respective provider by the first author

**Fig. 1:**
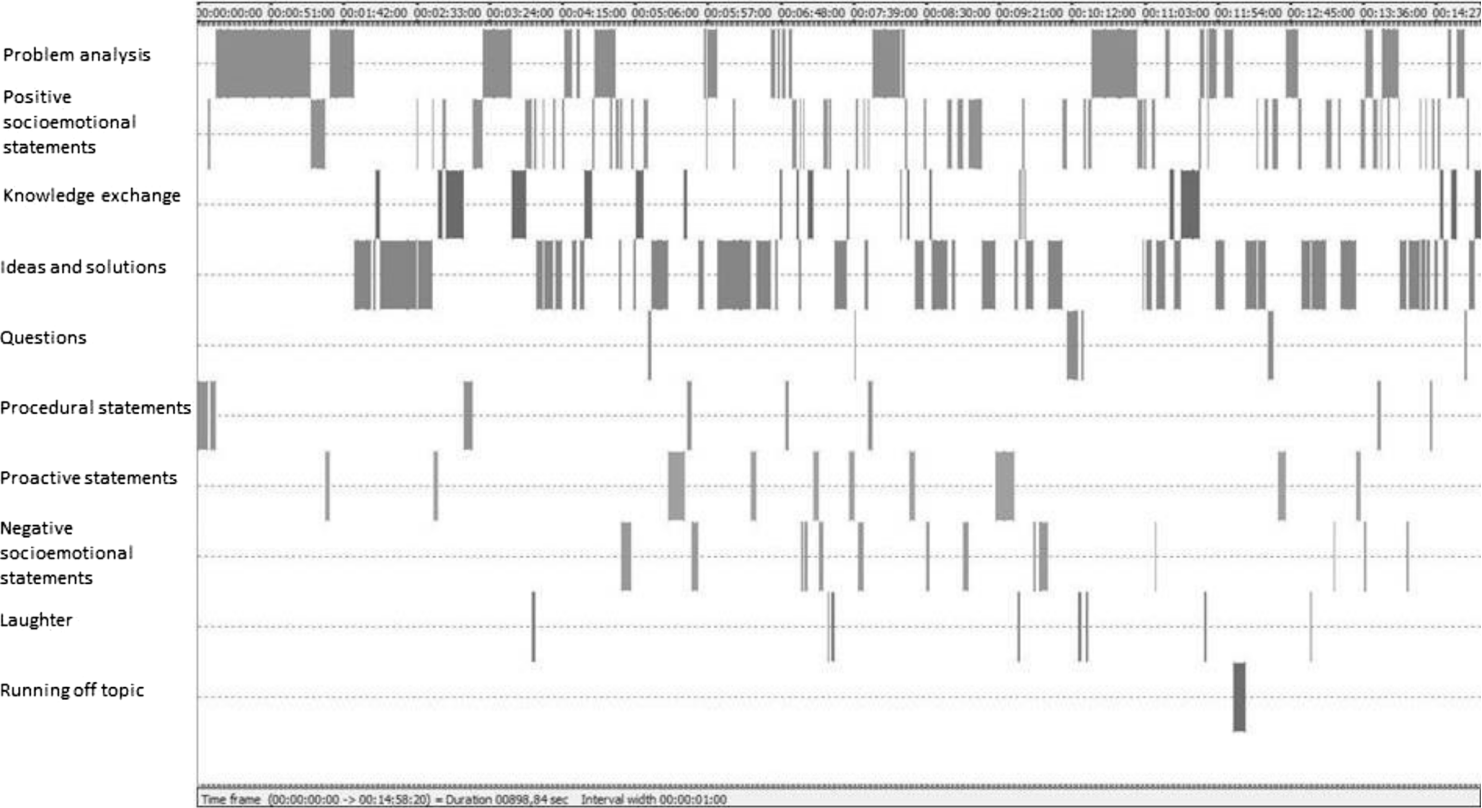
Screenshot from INTERACT software (Mangold, 2010), showing temporal sequences of coded team interaction behaviors at the beginning of a team meeting. Behavior onset and offset times are indicated in hours, minutes, seconds, and frames. The “participant” column indicates which person is talking at each behavioral event. The “code” column shows annotations with the act4teams coding scheme (e.g., Kauffeld & Lehmann-Willenbrock, 2012)

To illustrate the type of data that can be generated from temporal interaction data when using coding software, Fig. 1 shows the screenshot of a stream of meeting behavior coded with the act4teams coding scheme using INTERACT software (please note that we do not intend to advertise this software in particular; it just happens to be the software which we currently use for our own interaction analytical research). By unitizing directly from the video, researchers can go back and directly play specific behaviors of interest. In our team problem solving example, if we decided to use the act4teams coding, we could go back and play specific instances where a team used problem statements, or replay all those instances where a team used solution statement or problem elaboration. Follow-up analyses could then start to distinguish between different types and qualities of these particular statements.

In Table 3, we provide an overview of the different software programs mentioned in this paper (GSEQ, INTERACT, The Observer XT) and their respective functionalities. Of note, the summary in Table 3 is focused on the pattern analytical options described in this paper, and there are additional functionalities of the different software options. In addition to the statistical analyses highlighted in Table 3, GSEQ, GridWare, INTERACT, and The Observer XT also provide possibilities for graphical visualization. GSEQ can plot behavioral data in simple time × behavior plots. INTERACT and The Observer XT offer more extensive visualizations. Both programs provide time-sequenced visualizations of coded data (see Fig. 1 for an example). In addition, both programs include a chart module for visualizing descriptive statistics (e.g., pie charts for conversation shares or frequencies of specific behaviors). For a complete list of each program’s functions and capabilities, please contact the respective provider.

As opposed to INTERACT and The Observer XT software, GSEQ software is free of charge; however, it requires already coded data (see references section for the Web link). The decision which software to use is often restrained by the financial budget at a researcher’s disposal, yet it should also be driven by the research questions of interest. Different components of the commercial software packages INTERACT and The Observer XT come at different prices. Both companies offer trial versions and customer support so researchers can make an informed decision which components are necessary for analyzing their research questions of interest. Of note, both INTERACT and The Observer XT are designed for Windows operating systems. The recent releases of Observer XT software will only run on a Windows 64-bit system. INTERACT will run on either a Windows 32- or a Windows 64-bit system, and will also work on a Mac computer when running Windows rather than the Mac operating system on the computer. Potential issues can be ruled out by obtaining advice from the provider and making use of a free trial version prior to deciding on a software solution.

## Detecting Patterns of Behavior in Temporal Interactions

The fourth step of the process is to select a pattern analytical method. That step begins by completing the coding and then deciding how best to quantify the desired temporal patterns in the data. We highlight three particular options here that have increasingly been applied in recent years: lag sequential analysis, pattern analysis, and statistical discourse analysis. Importantly, each of these methods leads to *quantitative* output regarding emergent interaction patterns in social interaction data. This differentiates them from more descriptive, qualitative methods for analyzing interaction patterns such as microethnography (e.g., Liu & Maitlis, 2014) or interpretive approaches (e.g., van Oortmerssen et al., 2015). In addition, we discuss visualization techniques that are suitable for exploring interaction data, for example prior to selecting one of the quantitative analytical methods for identifying interaction patterns. As we will elaborate below, the decision which method to choose depends on the complexity of the interaction data at hand, as well as the study hypotheses or research questions of interest, respectively.

### Data Complexity

Depending on the unitizing decisions when studying interaction processes as actual behaviors (see Table 2 for different examples), researchers tend to have very large data sets on their hands. This concerns both the dimensionality of the data (e.g., considering 44 types of team problem solving behaviors; Kauffeld & Lehmann-Willenbrock, 2012) and the number of behavioral events (e.g., 43,139 verbal behaviors in a sample of team meetings; Lehmann-Willenbrock et al., 2016). For our sample research question introduced earlier, we would likely want 30 or so group interactions (e.g., team meetings), in order to pool data at the team level later, and in order to code using a comprehensive team decision-making coding scheme. It should be noted that we are essentially working with a multilevel model with individual behaviors being potentially mapped onto group level interactive processes. Thus, recommendations for multilevel models apply (i.e., rules of thumb for sample size; e.g., Maas & Hox, 2005).

Using a fine-grained coding scheme, observing a 1-h meeting of a single team and focusing on verbal behaviors (e.g., the act4teams coding scheme; Kauffeld & Lehmann-Willenbrock, 2012) typically already yields over a thousand behaviors. At the team level, researchers easily have tens of thousands of data points on their hands (e.g., Lehmann-Willenbrock, Meinecke, Rowold, & Kauffeld, 2015). Even more data points result when researchers combine observational methods such as coding the verbal communication with other observational tools, such as sensor badges for automatically detecting multimodal behavior (e.g., voice frequency, posture shifts, or gesture movements; for a discussion on combining such approaches, see Lehmann-Willenbrock et al., forthcoming; Lewis, Zamith, & Hermida, 2013). Thus, when choosing how to analyze the data and which method to choose for identifying possible interaction patterns, both the nested nature of the data and the sheer volume of data in the sample must be considered.

### Choosing an appropriate method for quantifying interaction patterns

As step four (i.e., selecting a pattern analytical method) unfolds, steps four and five merge a bit as the decision of the analytical method leads directly to the running of analyses and interpreting the results. We highlight three prominent methods for quantifying interaction patterns in social interaction data here. The choice for each method should be guided by the research questions or hypotheses that a researcher wishes to examine in the observed interaction data. Not all methods are suitable for all research questions. Hence, guided by the research question(s), researchers need to consider which types of interaction patterns they are interested in, which method would allow them to investigate these, and to what extent their data is suitable for the respective method. We provide examples for each method as well as key decision points for each method in the sections that follow. Table 4 summarizes the different methods and indicates the types of research questions that can be addressed by each method. For a detailed critique of the benefits and shortcomings of various methods for identifying temporal patterns in interaction data, we recommend Chiu and Khoo (2005), Herndon and Lewis (2015), or Waller and Kaplan (2016).

**Table 4:** Quantitative methods for analyzing temporal patterns in interaction data

Method	Approach	Types of research questions
Lag sequential analysis (e.g., Bakeman & Quera, 2011)	Tests whether observed transitions between specific behaviors in the data are statistically meaningful	Does behavior A trigger behavior B, C, or D? Which behaviors A, B, or C increase the likelihood for behavior D? Which behaviors A, B, or C can inhibit behavior D? How do patterns or cycles of behaviors A, B, and C emerge in the data? Who responds to whom? How does social influence emerge, based on speakers’ reactions to one another?
Pattern analysis (e.g., Magnusson, 2000)	Detects non-obvious or hidden temporal patterns among behaviors	Which behaviors are temporally related to one another (that do not necessarily follow one another immediately in time)? Which clusters of temporally connected behaviors emerge from the data? How complex are the detected interaction patterns?
Statistical discourse analysis (e.g., Chiu & Lehmann-Willenbrock, 2016)	Dynamic multilevel, time-series modeling of (1) pivotal actions that create breakpoints, (2) effects of previous actions on target actions, and (3) influences at multiple levels (conversation turn, time period, individual, group, organization, etc.)	Which behaviors radically change subsequent interaction processes, creating breakpoints and different time periods in the observed interaction data? How do recent behaviors affect the likelihoods of specific actions at each given turn of talk (or utterance or episode, etc.)? How do multilevel explanatory variables (e.g., individual dispositions and attitudes or team context variables) affect the likelihoods of specific behaviors? How does the strength of these explanatory links change over time?

### Lag Sequential Analysis

We begin our overview of available methods for quantifying emergent interaction patterns with lag sequential analysis, which is perhaps the most intuitive approach to testing how behaviors within an interaction stream influence one another. As summarized in Table 4, lag sequential analysis is suitable for testing hypotheses about which specific behaviors trigger which other specific behaviors in the data. To do so, lag sequential analysis provides information about whether observed behavioral sequences in the data are statistically meaningful.

For example, for the types of team interactions illustrated earlier in Fig. 1, we might test the hypothesis whether questions trigger novel ideas within the temporal flow of team interactions. Lag sequential analysis (e.g., Bakeman & Quera, 2011) determines whether a sequence of behavior that occurs in an interaction data set is meaningful (i.e., above chance). To do so, researchers first need to create a matrix that contains the frequencies of all interaction sequences in the data (e.g., how many times was the behavior “question” followed by “new idea” overall in our data set). So-called first-order transitions occur where one statement directly follows the previous one (Lag1); second-order transitions occur when a statement is followed by the next-but-one statement (Lag2); and so forth. Separate matrices need to be created for each Lag. Next, we can compute transition probabilities by dividing the cell frequencies by the cell sums. These probabilities indicate the likelihood that a specific behavior (e.g., “new idea”) is triggered by a given behavior (e.g., “question”) in the data.

Importantly, because transition probabilities are confounded with the base rates of the events that follow, a high transition probability does not necessarily indicate an above chance transition frequency. Moreover, some behaviors are typically much more frequent than others, which means that a substantial number of coded events are typically required in order to meaningfully interpret sequential analysis findings. The minimum number of behavioral events depends on the number of lags under investigation as well as the number of observational codes contained in the coding scheme (see Bakeman & Gottman, 1986, p. 149, for a formula to calculate the minimum number of events).

**Fig. 2:**
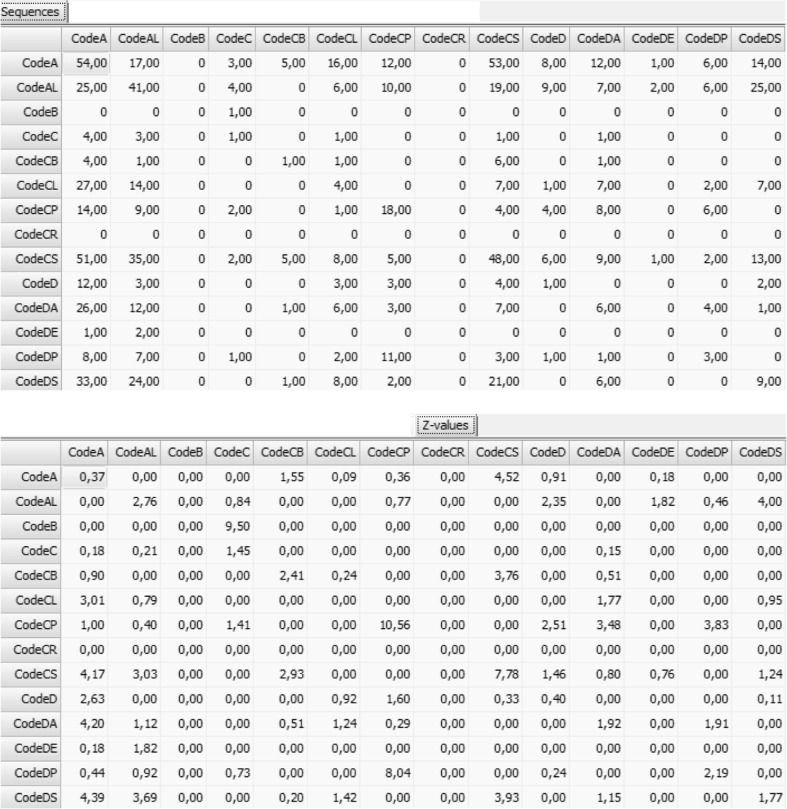
Partial screenshots of a Lag1 sequential analysis for a sample of 30 team meetings, generated with INTERACT software

To examine whether the observed transition probabilities are statistically meaningful, researchers can use the *z*-statistic as a statistical check (Bakeman & Gottman, 1997). Because this statistic is based on the normal distribution, values higher than 1.96 (or lower than −1.96) are statistically significant. Lag sequential analysis can be obtained as a component of INTERACT software or with The Observer XT. However, lag sequential analysis can also be performed using the freely available software GSEQ (Bakeman & Quera, 2011). Data coded in INTERACT or The Observer XT software can easily be converted into a GSEQ-compatible format (Bakeman & Quera, 2007). Figure 2 shows an example of a lag sequential analysis for a sample of 30 team meetings (sampled from Lehmann-Willenbrock & Allen, 2014). The upper section shows the number of behavioral transitions observed for each pair of behavior in the data set (e.g., “CodeCS” is followed by “CodeA” 51 times). The lower section shows the *z* values for each behavioral sequence. Any *z* value larger than 1.96 indicates statistical significance (e.g., the sequence “CodeCS”-“CodeA” is a statistically significant sequence; *z* = 4.17).

With a focus on specific behavioral linkages rather than general pattern complexity or duration, several studies have used lag sequential analysis to gain insights into team temporal dynamics. For example, we showed that temporal patterns of humor and laughter in real organizational team meetings were linked to higher team performance, both cross-sectionally and longitudinally (Lehmann-Willenbrock & Allen, 2014). Moreover, Kauffeld and Meyers (2009) used lag sequential analysis to identify statistically meaningful patterns of solutions and idea generation, as well as cycles of complaining behaviors. Another study showed how teams’ verbal patterns of complaining versus proactive behavioral patterns were linked to nonverbal behavioral indicators of emergent group mood during team meetings (Lehmann-Willenbrock et al., 2011). Another study used lag sequential analysis to identify the role of procedural/structuring behaviors during the team interaction flow in a sample of regular team meetings, and found that these behaviors can help inhibit dysfunctional behaviors such as complaining (Lehmann-Willenbrock, Allen, & Kauffeld, 2013).

In addition to these previous applications in the team literature, a small but growing research has also applied lag sequential analysis to the study of leader-follower interaction patterns in organizational settings. One study examined leader-follower interaction processes during 48 annual performance appraisal interviews and revealed reciprocal interaction patterns, such that supervisors’ relation-oriented statements triggered active employee contributions and vice versa (Meinecke et al. 2017). Another study used interaction coding and sequential analysis to show how solution-oriented leader behavior can trigger functional team interaction patterns and inhibit dysfunctional team member behavior such as complaining or criticizing others (Lehmann-Willenbrock et al., 2015). Compared to the team process literature, examples of quantitative behavioral interaction analyses in the leadership context remain rather sparse. We hope to see much more of this type of research in the future, given the wealth of opportunities inherent in such analyses, and the relatively easy interpretation of lag sequential analysis in terms of immediate behavioral triggers and/or inhibitors.

### Pattern Analysis

Pattern analysis is essentially a data mining technique that can identify “hidden” behavioral patterns, and as such is particularly suitable when researchers do not have a priori hypotheses about which specific behaviors will follow which other specific behaviors. As summarized in Table 4, pattern analysis is suitable for investigating exploratory research questions about non-obvious or hidden temporal patterns among behaviors. Whereas lag sequential analysis is suitable for testing hypotheses about specific behavioral linkages, pattern analysis takes a more holistic view and searches for patterns that are not obvious by merely looking at the data (Aldenderfer & Blashfield, 1984; Romesburg, 1984). As such, pattern analysis is a helpful exploratory method for detecting temporal patterns of behavior that are less “clean” than the immediate sequences of behavior that are typically examined by means of lag sequential analysis (see Magnusson, 2004, for a detailed discussion of the distinction between obvious versus hidden patterns). In other words, pattern analysis may reveal meaningful temporal connections between behaviors that are interrupted by (sometimes multiple) other behaviors. This would not be possible with the typical lag sequential approach, which looks for connections between behaviors that are adjacent (lag1) or at least close (lag2, lag3, etc.) to one another within the interaction stream.

Researchers can choose to set several parameters in order to guide the overall pattern detection process. For example, previous research using pattern analysis has focused only on those patterns that occur at least three times within an interaction, or to only highlight a pattern if the probability that it occurred above chance in the data is at least 95% (e.g., Sohrab, 2014). Such decisions can be necessary when pattern analysis would otherwise yield too many patterns to allow meaningful interpretation of the data.

In terms of current and up-to date software, pattern analysis can also be conducted using the p.a.t.t.e.r.n component in INTERACT software (Mangold, 2010; see Meinecke & Lehmann-Willenbrock, 2015, for a detailed application example). Similar to Magnusson’s (2000) earlier work, this method accounts for the temporal order, duration, and relative position of behavioral events. The underlying algorithm is based on Ward’s cluster analysis method (e.g., Aldenderfer & Blashfield, 1984; Romesburg, 1984). Starting with each type of behavior in its own cluster, the algorithm continues to merge clusters until it reaches one cluster that contains all coded behaviors. The first cluster is based on cases with the lowest squared Euclidean distance. While gradually adding cases to each cluster, the algorithm tracks the average similarity of the emerging cluster, first merging cases that increase the sum of squared deviations within a cluster. The emerged clusters are ordered according to the relation strength of each behavioral pattern.

**Fig. 3:**
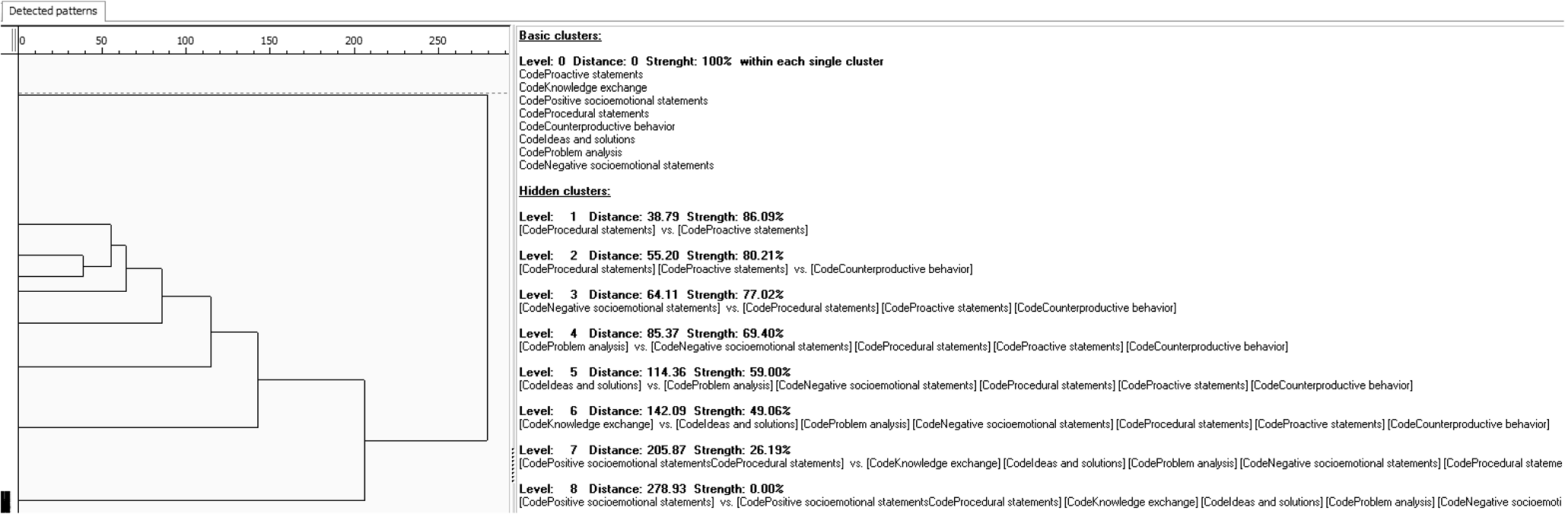
Pattern analysis for one sample team meeting, generated with INTERACT software

As an illustration, Fig. 3 shows an example of a pattern analysis for one entire team meeting, coded with the act4teams coding scheme (sample team meeting from Lehmann-Willenbrock & Allen, 2014). In this example, the strongest behavioral cluster concerns patterns of procedural and proactive statements. However, other clusters appear to have emerged (e.g., socioemotional statements) that could be interpreted, if desired. Because the detected patterns are highly dependent on the data and can only be interpreted in context, the researcher needs to decide whether a cluster is meaningful or not (Mangold, 2010). Yet, such an inductive can be very useful for exploring temporal patterns when there are no a priori hypotheses regarding specific types of behavior. For example, pattern analysis can reveal how the overall patterns of behavior change across different phases of a team meeting (Meinecke & Lehmann-Willenbrock, 2015).

Pattern analysis has successfully been applied by researchers studying temporal interaction patterns in the context of team performance. In particular, Zijlstra et al. examined the interaction of swift-starting aviation teams in a flight simulator and found that early interaction patterns were linked to team effectiveness (Zijlstra et al., 2012). Teams who showed higher performance had temporal patterns that were more reciprocal, more stable in duration, and more stable in complexity than those of the less effective teams. Hence, for the context of swift-starting teams, their findings point to the relevance of the first moments of interaction. Similarly, findings from nuclear plant teams during a crisis simulation showed distinct differences in the temporal interaction patterns of high versus low performing teams (Stachowski, Kaplan, & Waller, 2009). Moreover, a recent study on airline crews in a flight simulator highlights the role of different interaction patterns for team performance during routine versus non-routine work situations, such that successful teams show more in-process planning behavior patterns during routine versus non-routine situations (but only up to a point, i.e., U-shaped relationship between planning behavior patterns and performance; Lei, Waller, Hagen, & Kaplan, 2016).

### Statistical Discourse Analysis

Lag sequential analysis and pattern analysis detect linkages between behaviors, and consider previous behaviors within the interaction stream as predictors for future behaviors. However, the occurrence of a particular behavior at any given point in an interaction can also be due to other explanatory factors, such as individual speaker characteristics or characteristics of the social context in which the interaction occurs. As summarized in Table 4, statistical discourse analysis (SDA; e.g., Chiu, 2008; Chiu & Lehmann-Willenbrock, 2016) is an innovative method for quantifying these different types of influences on behavior within interactions. For example, imagine that in addition to the team interaction data illustrated in Fig. 1, you have also gathered survey data on different characteristics of the team members (e.g., demographic data, a personality measure, and a team climate survey). Hypotheses regarding the likelihood of contributing a new idea might now become more complex. In addition to previous behaviors within the interaction stream (e.g., questions), idea occurrences could also depend on the organizational tenure of the speaker, on the speaker’s level of extraversion, and on the climate for innovation at the team level. Moreover, there may be several effects of time, such that (1) ideas are generally more likely in later phases of the conversation, (2) the explanatory value of personality for idea occurrences is stronger in earlier phases of the conversation, and (3) the effect of preceding questions is significant at Lag3 prior to the idea occurrence, but not at Lags 1 and 2. Such complex explanatory models require sophisticated statistical models that can incorporate time-series analysis and multilevel modeling.

SDA can address this need as well as overcome a number of shortcomings of earlier methods such as lag sequential analysis. Yet, it is not always the preferred method; rather, as with the previously discussed methods, the decision for or against SDA should be guided by the research question(s). SDA should be considered when a research question about interaction patterns requires the inclusion of predictors not only at the behavioral event level (i.e., lag sequential or pattern analysis), but also at multiple other levels.

SDA deals with challenges involving data, dependent variables, and explanatory variables and has been applied to a number of different interaction contexts (for an overview, see Chiu & Lehmann-Willenbrock, 2016). Rather than predicting behavior only by preceding behavior at different lags (cf. lag sequential analysis or pattern analysis), SDA can simultaneously model the influence of multilevel explanatory variables on behavior. For example, when predicting the likelihood of positivity behavior in team interactions, this method can simultaneously model the effects of prior problem and solution statements, prior positivity statements, turn-taking behavior, interaction effects between these different variables, the overall discussion share of each individual, and the company to which each observed team belonged (Lehmann-Willenbrock et al., 2016). A way to think of this method is to imagine that each coded behavior is accompanied by all of the variables that are attached to each speaker (e.g., surrounding time period; individual demographics, personality, work attitudes; team level characteristics such as team size; or organization-level characteristics). Hence, each behavioral sequence (i.e., what behavior B follows a given behavior A?) can be predicted by the preceding behavior A (at different lags) and/or by explanatory variables at higher levels (e.g., time period, individual, team, organization).

In addition to painting a more comprehensive picture of different influences on behavior within temporal interactions, SDA also shows how much variance is explained at each level. For example, in the study on positivity in team interactions, individual characteristics and the surrounding organization only accounted for 8% of the variance in observed positivity (Lehmann-Willenbrock et al., 2016), which again underscores the need to move away from static individual-level research and toward temporal behavioral processes. Due to space limitations, we will not elaborate on the statistical details of this method here, but we strongly encourage organizational researchers who are interested in combining explanatory variables at the behavioral interaction level with explanatory variables obtained from individual, dyad, or team survey variables to consider this approach.

In sum, the choice of method for modeling temporal patterns of behavior (e.g., lag sequential analysis, pattern analysis, or SDA) should always be driven by the research question at hand. SDA has a number of advantages and allows simultaneous modeling of multilevel influences on behavior within an interaction stream. Yet, this method also requires substantial computational effort. For simpler hypotheses regarding patterns of behavior and influences of prior behaviors rather than individual/team/other context characteristics, simpler methods are suitable and often easier to implement.

### Visualization Techniques

In addition to the quantitative methods outlined above, there are a number of techniques for visualizing interaction patterns. These can be helpful for exploring trends in the data prior to conducting quantitative hypothesis testing, and often also include possibilities to quantify the visual information. With a focus on tools for facilitating pattern recognition, we highlight two visualization techniques here. First, *recurrence plots* illustrate those points in time where a system revisits an earlier state (Marwan, 2008; Marwan, Romano, Thiel, & Kurths, 2007). When applied to temporal interaction data, a recurrence plot is a graphic representation of the square matrix of those times when a behavioral state reoccurred in the interaction (e.g., once a problem statement occurred, when does the interaction move to another problem statement). The columns and rows in the matrix represent a specific pair of behaviors (e.g., problem–problem).

The hidden patterns and nonlinearities that can be detected using recurrence plots can also be quantified (i.e., recurrence quantification analysis; for different quantification options and measures, see Marwan, 2016). A discussion of recurrence quantification in the context of social interaction data can be found in Gorman, Cooke, Amazeen, and Fouse (2012) as well as Knight, Kennedy, and McComb (2016). There is freely available software for conducting this type of analysis on coded temporal interaction data (e.g., Belaire-Franch, Contreras, & Tordera-Lledó, 2002; for an overview of freely available and commercial software options, see Marwan, 2016). Moreover, the basic principle of mapping recurrent behaviors can also be applied to recurrent behavioral sequences (e.g., problem–solution) rather than recurrences of singular behaviors (Quera, 2008).

A second option for visualizing interaction patterns concerns *state space grids.* This method visualizes the relationship between two behaviors that are synchronized in time (Lewis, Lamey, & Douglas, 1999). State space grids can be generated using an analysis package in INTERACT software or by means of GridWare, which is freely available for download (Lamey et al., 2004) and also compatible with Noldus’ The Observer software.

In the context of social interactions, state space grids are a helpful tool for visualizing dependencies among simultaneously coded data. Simultaneous coding means that a behavior is associated with several codes. For instance, this would be the case when considering co-occurrences of specific speakers and specific behaviors, such as the dependency of the behavior “Question” on speaker “A” in our earlier example shown in Fig. 1.

**Fig. 4:**
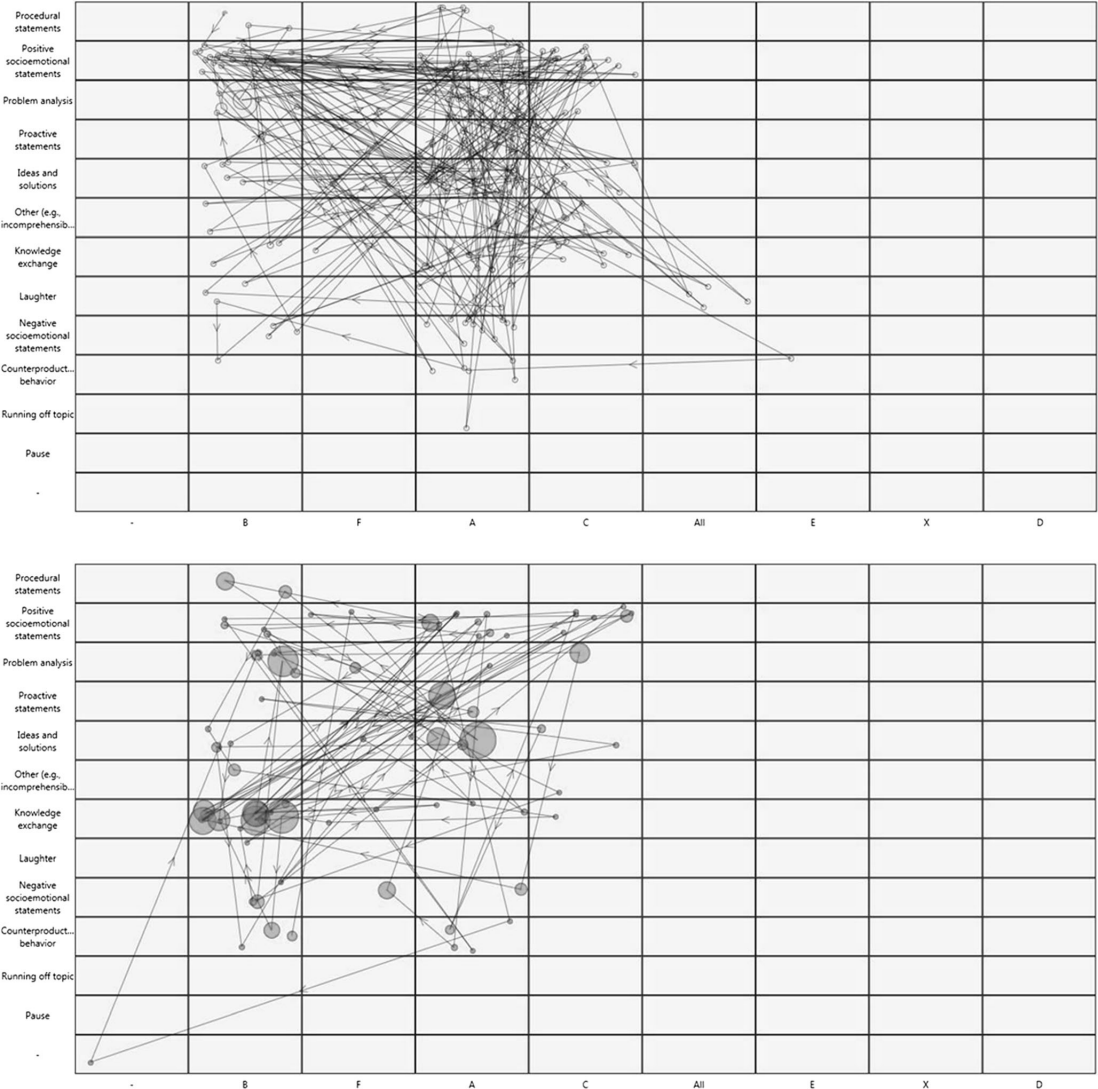
Two state space grids for the first 15 min (upper part) and the final 15 min of a team meeting, generated with INTERACT software. The respective x-axis depicts the different team members (e.g., “B”). The respective *y*-axis depicts different types of communicative behaviors

To illustrate what such a state space grid might look like, we provide an example generated from one team meeting in Fig. 4 which was coded with the act4teams coding scheme (sample taken from an earlier data set, Lehmann-Willenbrock & Allen, 2014). The upper section of Fig. 4 plots the interaction behavior exhibited by each team member (e.g., “B”) during the first 15 min of this meeting. The lower section of Fig. 4 shows the interaction behavior by each team member during the last 15 min of the same meeting. As illustrated by these two state space grids, the interaction behavior of this team changed considerably over the course of their meeting. For example, whereas team member B showed a range of behaviors with a focus on problem analysis statements early in the meaning, he spends most of his conversation shares on knowledge exchange later on in the meeting. Moreover, the different state space grids for the different time periods within the meeting showcase how conversation shares as well as the overall behavioral configuration in this team shifted over time.

The trajectories plotted on state space grids can also be quantified for hypothesis testing (Hollenstein, 2013). For example, one study used state space grids to examine coach-athlete interactions and found significant differences in interaction variability, behavioral patterns, and the sequencing of coach behaviors in high- versus low-performing sports teams (Erickson, Côté, Hollenstein, & Deakin, 2011). We can only speculate what such applications might look like in organizational settings at this point. However, future research on interactions in organizational teams might utilize state space grids to visualize the interaction dynamics in high- versus low-performing teams. Moreover, state space grids can be used to visualize lagged events, which can facilitate interpretation of other pattern detection approaches such as lag sequential analysis (see also Meinecke & Kauffeld, 2016).

Exploratory visualization methods such as recurrence plots or state space grids can be very helpful for tackling the “bigness” of interaction analytical data, which we elaborated on earlier. They can provide ways to explore this rich data in non-intuitive ways that would otherwise not be accessible to the human eye. In the ideal case, such visualization methods can then pave the way for novel research questions, which reflects an inductive research paradigm (for a discussion of the benefits of inductive research, see Spector, Rogelberg, Ryan, Schmitt, & Zedeck, 2014). Insights from this inductive phase that are channeled into a priori hypotheses can later be tested deductively with quantitative pattern analytical methods (such as pattern analysis, lag sequential analysis, or SDA). Combining these different approaches means reaping the benefits of both inductive and deductive research, with the goal of maximizing research insights obtained from temporal interaction data.

## Key Takeaways

The purpose of this *Methods Corner* article was to provide recommendations and a tutorial for observing and analyzing behavioral, temporal interaction patterns in organizational settings. Following our overview of possible approaches for observing and analyzing such behavioral patterns, we want to highlight a number of key takeaways in the hope that these will inspire more scholars to embrace such methods and leverage their potential for their own research:

1. When you talk about behavior, please study actual behavior.

This first takeaway may seem obvious and straightforward, and readers may wonder why it even needs to be raised here. Yet, through our own experience, through talking with other colleagues involved in this kind of methodology, and in developing this article, we noticed that this point absolutely needs to be emphasized. Actual behavior is chronically understudied in psychology, despite psychology’s scientific aim to explain human behavior (for a detailed critique, see Baumeister et al., 2007). As we searched for examples to include in our paper, we were surprised to find an abundance of paper titles and abstracts indicating the study of “field samples,” “actual behavior,” and “temporal interaction data” that nevertheless relied on student samples, reports of behavior, and static rather than temporal measures. Although there are some great examples of rigorous temporal interaction data and analysis, we recommend caution when searching the field for examples to follow and consider the criteria put forth in this methods corner when proceeding to engage in this type of research.

Organizational psychology is no exception to the general criticism of lacking behavioral research (Baumeister et al., 2007), as the vast majority of empirical work continues to rely on survey methodology that captures *proxies* of employee behavior, rather than observing actual behavior (e.g., Carpenter, Berry, & Houston, 2014; Fida, Paciello, Tramontano, Fontaine, Barbaranelli, & Farnese, 2015; Kehoe & Wright, 2013; Petrou, Demerouti, & Schaufeli, 2016). Although proxies or interpretations of employee behavior are important and an entire epistemology within psychology and the organizational sciences relies upon these approaches (i.e., survey studies and the cognitive psychology tradition; Gardner, 2008), in many cases, the visible/audible behavior is closer to the phenomenon of interest (Baumeister et al., 2007).

For example, when studying participation in decision-making, the observed behavior (i.e., frequency of statements in a group context) may vary greatly from the post hoc feeling of such behavior (i.e., retrospective perceptions of participation of decision-making). Additionally, rather than relying on a single individual’s interpretation, the approaches described here require multiple raters to rate the actual behavior and that those raters agree. Yet, the field is clearly moving toward embracing more behavioral research and accounting for the temporal dynamics that characterize many interaction phenomena in the workplace. The outlook for modeling temporal interaction dynamics in organizational settings is bright, but several issues must be considered in order for scholars to move forward from the continued calls for such research (e.g., Kozlowski, 2015).

2. Start embracing available methods rather than (repeatedly) calling for future research endeavors for investigating interaction patterns.

Second, scholars should acknowledge and build upon the already existing studies that use the methods and tools discussed in this methods corner. A number of researchers and scholars have been using dynamic social interaction analysis techniques for quite a few years, particularly in research on team processes but increasingly also when considering leader-follower dynamics in organizations (e.g., Kolbe et al., 2014; Lei et al., 2016; Meinecke et al., 2017). These studies provide a nice base from which researchers new to these methods can draw considerable methodological advice and guidance as they study new areas using similar methods. Yet, scholars need to take active steps in this regard.

Today’s graduate students are tomorrow’s methodological innovators. One way to embrace available methods for observing and analyzing interaction patterns then concerns finding ways to include such methods in graduate student curricula. For example, many graduate programs in the organizational sciences offer a group dynamics class, either as a requirement or as an optional seminar course. Including temporal dynamic interaction analysis and processes as part of that course seems a meaningful place to initiate interest among graduate students. Additionally, as graduate students increase their interest in studying groups/teams, encouraging them to consider dynamic temporal interactions will help introduce the methodology to them and others involved with their projects. Further, for researchers already studying groups and teams, simply adding a camera, strategically placed in the lab or team meeting room, will provide an easy way to get the very data needed by students to begin doing temporal dynamic research. Further, it should be noted that the Society for Industrial and Organizational Psychology’s recent guidelines update included “Groups and Teams” as a core content domain thereby encouraging the training of future organizational scientists who have a level of competence in this domain. Thus, including methodologies at the forefront of the study of groups and teams will help students prepare for their future careers.

3. Observe and analyze interaction patterns among employees in the field.

Third, many current research efforts using temporal interaction data and analyses rely on laboratory or simulation data. Although lab settings are excellent contexts for capturing this kind of data in a controlled context, field research in less controlled contexts is needed. For example, lab research on leadership in team meetings using students ultimately cannot move beyond proxies for the actual organizational context of interest where more high-stakes decisions and interactions occur. We acknowledge that field research is complicated and that only increases when seeking to gather more comprehensive data, like audio or video. However, we see the benefits as greatly outweighing the effort and hope that scholars will continue advance the science by building partnerships with organizations that will allow such field research (see also Rosen, Wildman, Salas, & Rayne, 2012).

4. As a journal editor or reviewer, be open to novel methodologies.

Fourth, a pragmatic issue associated with this type of research is encouraging journal reviewers and editors to be more open to methods that they are not personally familiar with and allow for such methods to push the field forward. Naturally, this point is not limited to the use of behavioral observation methods and pattern analytical methodology but rather applies more broadly. Although researchers are beginning to investigate phenomena with temporal interaction data and analyses, many editors and reviewers are not familiar with these procedures, making the review process particularly difficult. Time and persistence on the part of the researchers to push their research using behavioral micro-processes in organizations will help to resolve this, but a greater empathy and consideration toward these types of methods would certainly assist in advancing the science.

5. Seek out interdisciplinary collaborations.

In addition to these issues, researchers aiming to model temporal interaction processes in organizations should really embrace interdisciplinary collaborations to move the methods and their own field of inquiry forward. For example, organizational psychologists should talk to communication scholars, who are experts at defining and studying phenomena at the micro-level and at observing real interactions (for an overview, see Keyton, 2017). Statisticians who are experts in connecting temporal interaction data to more static individual or team level variables are needed, particularly those who can handle big data sets and the associated multilevel models. Finally, looking beyond the traditional social sciences (e.g., psychology, communication, management, and sociology) and seeking out active collaboration partnerships with computer scientists who are experts at automatized behavior detection is where the field needs to push next (see also Lehmann-Willenbrock, Hung, & Keyton, in press; Waller & Kaplan, 2016).

In sum, our hope is that more researchers studying groups, teams, and leadership in organizations will be inspired to investigate phenomena of interest as they emerge through dynamic temporal and social interaction where real observed behavior is captured. The methods described here are powerful tools for truly leveraging such data.
